# Assessment of Flue Gas Emissions in Faisalabad's Textile Industry: A Comparative Analysis of Fuel Types

**DOI:** 10.1155/ianc/9955400

**Published:** 2025-08-29

**Authors:** Muhammad Ahmad Akram, Khalid Mahmood Zia, Saima Rehman, Shazia Tabassum

**Affiliations:** Department of Chemistry, Government College University, Faisalabad 38030, Pakistan

**Keywords:** air quality index (AQI), emission control system (ECS), flue gases, fuel types, oxides of nitrogen (NO_x_), oxides of sulfur (SO_x_), PEQS, smoke opacity

## Abstract

This study examines the flue gas emissions originated from various fuel types used in the textile industries of Faisalabad, Pakistan, and their compliance with the Punjab Environmental Quality Standards (PEQS), Pakistan. Data from 109 textile factories revealed significant emission variations based on fuel types. Natural gas was identified as an eco-friendly fuel, with emissions far below the PEQS limits (CO: 334.8 mg/Nm^3^, SO_2_: 175 mg/Nm^3^, NO_x_: 692 mg/Nm^3^). Moderate fuels such as corncob, wood, and coal exhibited emissions that slightly exceeded PEQS limits but could be mitigated by adopting advanced emission control systems. In contrast, cloth waste exhibited the highest emissions, significantly exceeding PEQS thresholds (CO: 2091.4 mg/Nm^3^, SO_2_: 2978 mg/Nm^3^, NO_x_: 487.5 mg/Nm^3^), followed by mixed fuels such as wood + cloth waste. Smoke opacity and flue gas concentrations were used to classify the fuels into eco-friendly, moderate, and acute impact categories. Findings underscore the urgent need for the adoption of cleaner fuels, enhanced emission control systems, and stricter regulatory enforcement to mitigate environmental and health impacts in Pakistan's textile sector. This research provides a framework for transitioning to sustainable industrial practices, aligning with global climate action and sustainable development goals (SDGs).

## 1. Introduction

Air pollution refers to the introduction of harmful substances into the atmosphere, exerting significant implications for the environment and its inhabitants [1]. Air pollutants are majorly introduced into the environment through vehicle emissions, industrial activities, and the combustion of fossil fuels [2, 3]. The key air pollutants include oxides of carbon (CO, CO_2_), sulfur (SO_x_), nitrogen (NO_x_), and volatile organic compounds (VOC_s_), along with particulate matter (PM) of different sizes, e.g., 2.5 μm–10 μm [4]. Air pollution is a widespread problem responsible for nearly 7 million premature deaths yearly [5]. The health effects of air pollution vary and range from asthma, eye irritation, cardiovascular diseases, to even lung cancers and lead to death [6, 7].

Flue gases, one of the major industrial contributor of air pollution, are released after the combustion of fuels in boilers, oil heaters, and other industrial processes [8]. These flue gas components can cause smog, acid rain, and respiratory illnesses [9]. Management to reduce these flue gas emissions is crucial to improve air quality and minimize environmental threats to citizens living near the industrial areas [10–12]. For this, flue gas analysis technology and emission control systems (ECS) have been introduced in many regions. Still, the application of these tools in textile industries of developing countries, e.g., Pakistan, has been limited. This study combines the results of advanced flue gas analyzers and US EPA methods to provide cutting-edge insights into flue gas emissions from various fuel sources.

Pakistan, being a country with a lot of textile industry, is highly vulnerable to the adverse effects of air pollution, and industrial cities such as Faisalabad, Lahore, and Karachi are the major contributors to the country's deteriorating air quality [7, 13]. Due to this, the entire province of Punjab began to experience smog phenomena during early winter months every year since 2016 to date [14].

Faisalabad is known as the “Manchester of Pakistan” due to its prominence in textile manufacturing and a significant portion of textile exports. Still, it is affected by severe air pollution because of its reliance on energy-intensive industrial processes. Steam boilers and oil heaters in textile industries emit a large amount of flue gases that negatively impact the environment and human health [15, 16]. Children and the elderly are most likely to suffer from respiratory diseases due to deteriorated air quality [17]. Therefore, it is necessary to investigate the root cause of this problem, which could be the fuel that industries use for energy production [18, 19].

Therefore, this study provides a fuel-specific analysis and categorization of air pollution concentrations, as there is a research gap related to the lack of comprehensive comparative studies of flue gas emissions from various fuel types in the textile industry and its compliance with the Punjab Environmental Quality Standards (PEQS). This study used different fuel types and recorded the flue gas emissions (CO, SO_x_, and NO_x_) from steam boilers and oil heaters in 109 textile industries in Faisalabad across a range of pure and mixed fuels, including natural gas, coal, wood, corncob, and cloth waste. A comparison was made between these emissions and the flue gas emission standard compliance status prescribed by the PEQS.

Moreover, this research also aligns with several sustainable development goals (SDGs), e.g., SDG 7 (Affordable and Clean Energy), SDG 9 (Industry, Innovation, and Infrastructure), and SDG 13 (Climate Action). By identifying eco-friendly fuels and promoting emission control strategies, the study will promote sustainable industrialization and will contribute to global climate action.

## 2. Materials and Methods

### 2.1. Description of the Study Area

**Figure 1:**
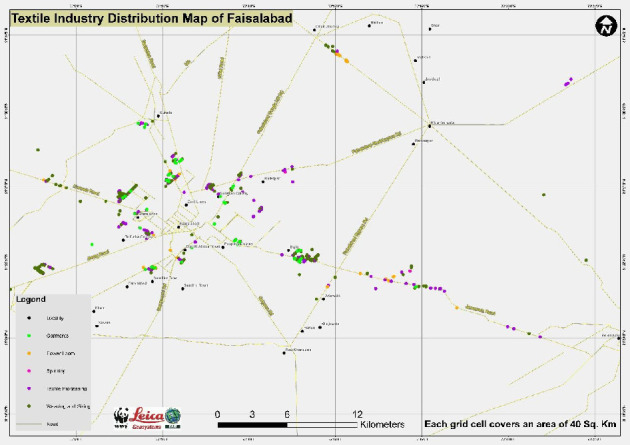
A map depicting the distribution of textile industries in Faisalabad, Pakistan (source: WWF, Faisalabad, Pakistan).

Faisalabad is located in Punjab, Pakistan (31.418715°N, 73.079109°E), which is famous for the textile industry. It covers approximately 195 km^2^ and lies between the Chenab and Ravi Rivers [20].  Figure 1 illustrates the industrial landscape of Faisalabad, providing a detailed breakdown of the regional distribution of industries.

### 2.2. Sampling Sites

**Figure 2:**
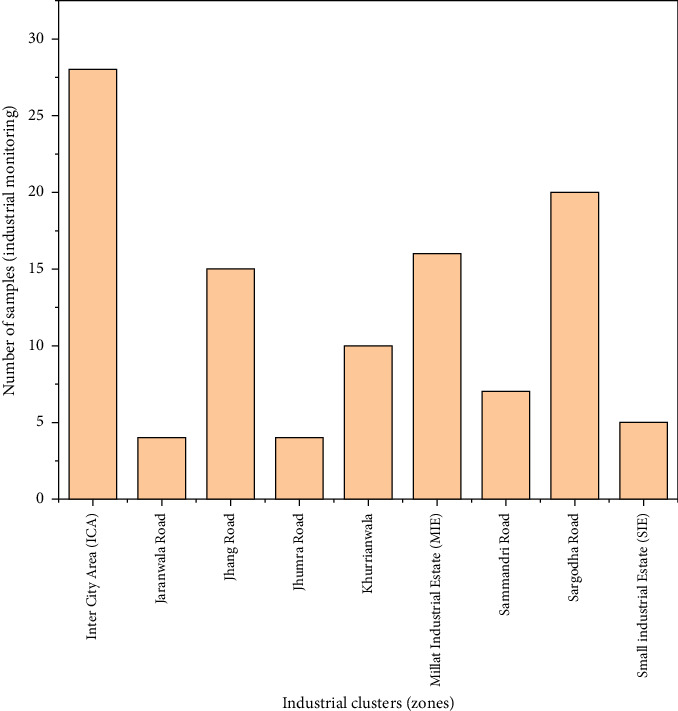
Sampling sites located in different industrial clusters (zones).

The flue gas emission sampling was conducted in nine industrial zones (109 sites) in Faisalabad, representing older clusters like Inter City Area (ICA), Jhang Road, Millat Industrial Estate, Sargodha Road, Sammandri Road, and Khurrianwala (pre-1970) and newer ones like Jaranwala Road, Small Industrial Estate, and Jhumra Road (post-2010). The older clusters represent traditional textile operations, while the new ones represent modern methods. In selecting these industrial clusters, we sought to reflect the diversity of textile activities, such as dyeing, sizing, processing, and printing. This approach included both high-emission and low-emission zones, representing small-scale and large-scale industrial operations. The site selection provides industrial diversity, technological variation, and operational scales to ensure a comprehensive representation. A detailed description of the sample size from each area is depicted in Figure 2.

### 2.3. Instrumentation

Standard test methods based on electrochemical sensors were used to measure the concentrations of flue gases such as oxides of sulfur (SO_x_), nitrogen (NO_x_), and carbon (Table 1). The flue gas analyzer (Testo 350 XLM) was used to monitor the flue gases [21, 22]. Various US EPA methods were adopted for sampling/monitoring and calibration [23], while smoke opacity was measured using the Ringelmann Scale, which is used for the visual method [24].

### 2.4. Equipment Calibration

The flue gas analyzer was calibrated according to the manufacturer's instructions to ensure accurate and reproducible measurements. The analyzer was calibrated with certified zero gas (pure nitrogen) for baseline accuracy and pollutant concentration. On-site calibration kits ensured real-time reliability, complemented by periodic quality control checks. Quality control checks with calibration gases were conducted at the start and end of each session to validate the equipment's stability.

### 2.5. Data Acquisition Process

The flue gas analyzer collects real-time data and flue gas composition via a sampling hose equipped with a particle filter to remove contaminants. The flue gas is drawn into the analyzer, where sensors detect the pollutant levels and composition. The data are then processed and displayed on the control unit. The Testo Easy emission software (version 2.9 SP1) was used to retrieve the data using Bluetooth mode and via an RS232 cable.

### 2.6. External Factors

The combustion process in the textile sector is influenced by industrial activities, weather conditions, and the type of fuel usage. Other factors like furnace design, fuel moisture, and fuel type impact combustion efficiency. Weather elements such as temperature, humidity, and air pressure also affect pollutant formation. These factors were considered during the flue gas monitoring process.

### 2.7. Data Analysis

The emission data of various fuel types were analyzed and compared. Based on their environmental impact, fuels were classified into eco-friendly, moderate, and acute categories (Table 2).

### 2.8. PEQS

The flue gas concentrations were compared with the PEQS. The PEQS for oxides of sulfur (SO_x_) and oxides of carbon are 1700 mg/Nm^3^ and 800 mg/Nm^3^, respectively. While oxides of nitrogen (NO_x_) are measured at 1200 mg/Nm^3^ for coal-fired plants, 600 mg/Nm^3^ is measured for oil-fired plants and 400 mg/Nm^3^ for gas-fired plants. For smoke opacity, it is 40% or 2 on the Ringelmann Scale or equivalent [25, 26]. The PEQS were notified by the Government of Punjab, Pakistan (https://epd.punjab.gov.pk/peqs).

### 2.9. Statistical Methods

The collected data underwent statistical processing, and flue gas concentrations and smoke opacity were summarized using descriptive statistics, including means, standard deviations, and ranges. All statistical analyses were performed using IBM SPSS Statistics (version 27.0.1.0), with a significance level set at *p* < 0.05, enabling reliable conclusions regarding emission variability and compliance with environmental standards.

## 3. Results and Discussion

### 3.1. Flue Gas Concentrations

Boilers and oil heaters generate flue gases along with smoke opacity from the combustion of various fuel types. These pollutants were measured and categorized based on their environmental impact. The concentrations of pollutants across the fuels are summarized in Table 3.

### 3.2. Smoke Opacity

Smoke opacity can be easily detected (visual observation) and can be reported by the observer. It quantifies the density of smoke particles in the air that can absorb the amount of light beam so that the light is obscured by smoke [27]. The Ringelmann Scale is used to measure smoke opacity visually. It is based on a series of charts that compare smoke density. Smoke numbers range from 0 to 5, with 0 representing no smoke (white) and 5 representing extremely dense smoke (black smoke) [28]. Smoke opacity indicates the combustion level and efficiency level of combustion sources like chimneys, engines, and vehicular engines [29].

Smoke opacity was measured for both pure and mixed fuels in the study. Natural gas (1.0 ± 0.000) has the lowest smoke opacity, making it the most eco-friendly fuel. Considering their relatively higher smoke opacity values, wood (3.6 ± 1.236), corncob (3.2 ± 1.303), and coal (3.8 ± 1.0387) were categorized as moderate fuels. Conversely, cloth waste (5.7 ± 1.272) and wood + cloth waste (5.5 ± 1.732) demonstrated the highest opacity levels, indicating poor combustion.  Table 4 summarizes the smoke opacity values and rankings for the tested fuels, highlighting significant variations in environmental impact.

**Figure 3:**
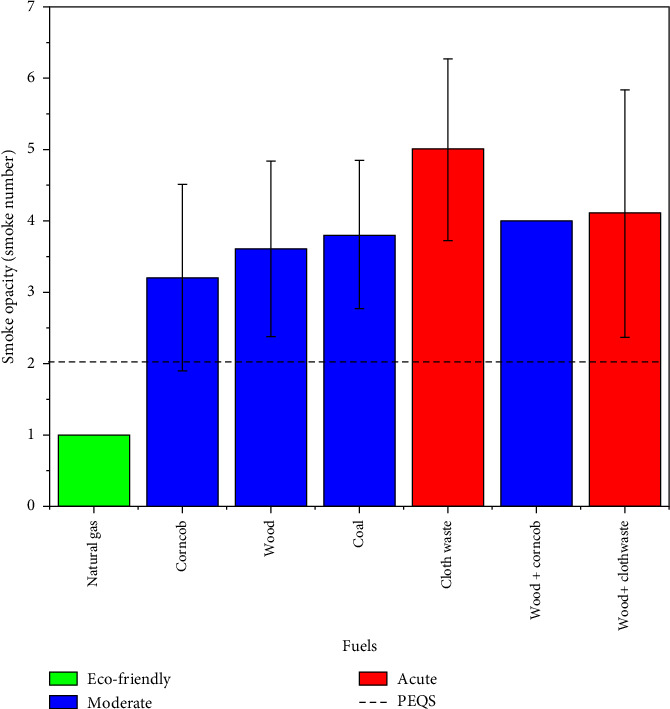
Smoke opacity levels for various fuels.

Natural gas exhibits the lowest smoke opacity (∼1.0), making it eco-friendly and fully compliant with the PEQS. Corncob, wood, and coal fall into the moderate category but exceed the PEQS thresholds. Conversely, cloth waste (especially wood + cloth waste) is the worst offender due to inefficient combustion and significant variability, as shown by the standard deviation bars in Figure 3. The dotted line represents the standard value by PEQS (RS 2.0).

### 3.3. Oxides of Carbon

Oxides of carbon, primarily carbon dioxide (CO_2_) and carbon monoxide (CO), are produced by combustion processes such as steam boilers and thermal oil systems. Carbon dioxide (CO_2_) concentration in flue gases depends on excess air and fuel carbon content [30]. There is a relationship between the amount of carbon monoxide (CO), the type of fuel used, and the significance of combustion [31]. The CO/CO_2_ ratio indicates combustion efficiency, with lower CO emissions signifying more complete combustion. When combustion temperatures decrease, carbon monoxide (CO) emissions increase, while carbon dioxide (CO_2_) emissions increase [32].

Among the tested fuels, natural gas (334.8 mg/Nm^3^) was found to be the most environmentally friendly and complied with the PEQS. Its superior performance is attributed to its high methane content, which ensures a clean and complete combustion process. However, biomass fuels such as wood and corncob can be controlled with proper ECS while slightly exceeding the PEQS limits (923.5–1132 mg/Nm^3^). Coal (1355 mg/Nm^3^) and industrial wastes such as cloth waste (2091 mg/Nm^3^) exhibit significantly higher emissions due to their complex organic structures and impurities, highlighting the need for more stringent controls or alternative fuel resources. Specifically, cloth waste is classified as an acute fuel because of its high synthetic content, which results in incomplete combustion and elevated carbon dioxide emissions. Mixed fuels like wood + cloth waste also fall into this category, as they combine the inefficiencies of their components.  Table 5 summarizes the CO values and rankings for the tested fuels, highlighting significant variations in environmental impact.

**Figure 4:**
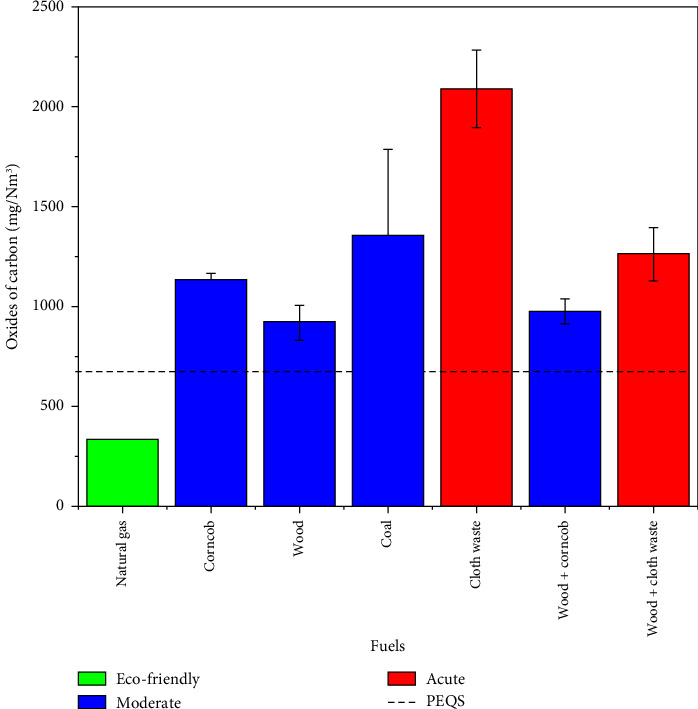
Comparison of carbon monoxide emissions across various fuel types relative to PEQS.

As illustrated in Figure 4, natural gas is the cleanest and most eco-friendly fuel, with low CO emissions and full compliance with PEQS (800 mg/Nm^3^), represented by the dotted line. Biomass fuels like wood, corncob, and their mixtures moderately exceed PEQS but remain manageable with proper ECS. In contrast, fossil fuels like coal and industrial waste fuels such as cloth waste have the highest emissions, posing significant environmental risks and requiring stricter controls or alternative solutions.

### 3.4. Oxides of Sulfur (SO_x_)

Oxides of sulfur (SO_x_), primarily sulfur dioxide (SO_2_), are significant pollutants produced during fuel combustion, particularly in sulfur-rich fuels like coal and industrial waste. Sulfur emissions depend on the sulfur content and combustion efficiency of fuels [33]. Coal particle size impacts the production of PM. Bituminous coal, lignite, and their blends were evaluated for combustion and emissions. Fly ash, sulfur dioxide (SO_2_), and nitrogen oxides (NO_x_) are three primary pollutants that coal combustion produces [34]. The data presented in Table 6 highlight the significant variations in sulfur dioxide (SO_2_) emissions across different fuel types. With emissions (175 mg/Nm^3^), natural gas stands out as an eco-friendly fuel, fully compliant with the PEQS. Biomass fuels such as corncob (1418 mg/Nm^3^) and wood (1354 mg/Nm^3^) are classified as moderate, as their SO_2_ emissions exceed the PEQS but can be managed with the implementation of an ECS.

Conversely, fossil fuels like coal (1902 mg/Nm^3^) and industrial waste such as cloth waste (2978 mg/Nm^3^) emit the highest SO_2_ levels due to their high sulfur content, placing them in the acute category. Mixed fuels exhibit variable behavior, with wood + corncob (1196 mg/Nm^3^) showing reduced emissions compared to pure biomass due to dilution effects during combustion. However, wood + cloth waste (2208 mg/Nm^3^) remains in the acute category, indicating the challenges posed by synthetic materials in industrial waste.

Wood + corncob emits fewer sulfur oxides (SO_x_) because alkali metals, such as potassium and sodium, react with sulfur during combustion to form stable sulfates instead of sulfur dioxide [35, 36]. Biomass fuels like wood and corncob inherently have lower sulfur content (< 0.5%) than coal (1%–3%) [37]. In contrast, cloth waste emits significantly higher SO_x_ levels due to synthetic materials that contain sulfur-based chemicals used in dyeing and finishing processes [38]. The detailed results regarding fuel types in comparison to oxides of sulfur (SO_x_) are presented in Table 6.

**Figure 5:**
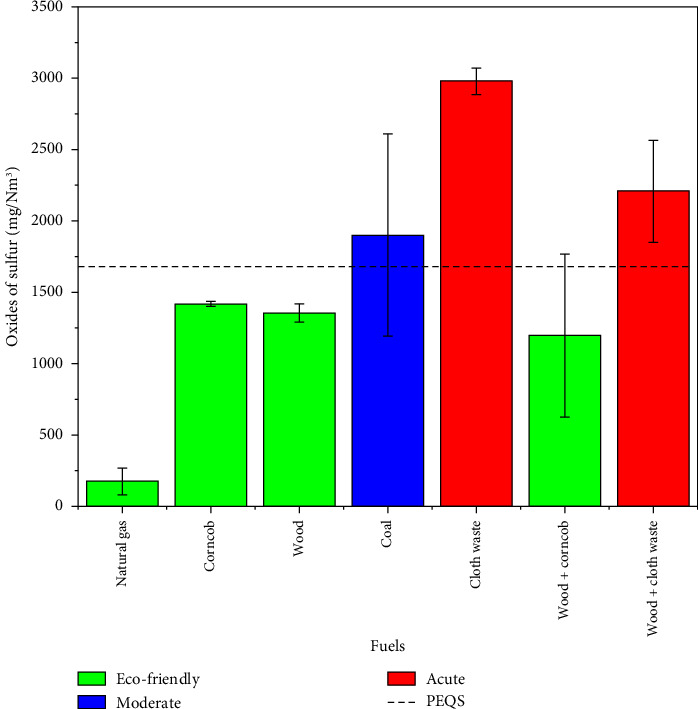
Comparison of sulfur dioxide emissions across various fuel types relative to PEQS.

Figure 5 illustrates that fuels are categorized based on sulfur dioxide (SO_2_) emissions and compliance with the PEQS, represented by a dotted line. Natural gas is considered eco-friendly compared to other fuels, as its emissions are well below the PEQS (175 mg/Nm^3^). Wood, corncob, and wood + corncob fall within the moderate category, slightly exceeding or within the PEQS. In contrast, coal (1900 mg/Nm^3^), cloth waste (2978 mg/Nm^3^), and wood + cloth waste (2208 mg/Nm^3^) are classified as acute, exceeding the PEQS. Moderate fuels need ECS, while acute fuels need stricter regulations.

### 3.5. Oxide of Nitrogen (NO_x_)

Oxides of nitrogen (NO_x_), such as nitrogen dioxide (NO_2_) and nitric oxide (NO), are significant pollutants formed during combustion processes. Various factors influence NO_x_ emissions, including combustion temperature, oxygen availability, and fuel nitrogen content [35]. It is challenging to predict oxides of nitrogen (NO_x_) emissions from coal-powered boilers due to complex combustion processes. SVR, LSSVM, and intelligent combinatorial algorithms have emerged as promising tools for modeling nitrogen oxide emissions [39].

Nitrogen in biomass fuels, such as proteins and amino acids, is primarily responsible for NO_x_ emissions during combustion. In high-temperature combustion, biomass with a high nitrogen content produces more NO_x_.  Table 7 explains the variation of fuel and flue gas emissions based on their measured values. Corncob (23 mg/Nm^3^) emits the lowest levels of NO_x,_ making it an eco-friendly fuel. Wood and wood + corncob emissions are moderate, with 317.33 mg/Nm^3^ and 331.16 mg/Nm^3^, respectively. Despite its clean combustion chemistry, natural gas (692 mg/Nm^3^) is categorized as acute. Similarly, the inefficient combustion of cloth waste (487.5 mg/Nm^3^) and wood + cloth waste (406 mg/Nm^3^) makes them acute fuels. Coal (206.16 mg/Nm^3^) emissions are categorized as moderate, but variations in combustion conditions could increase emissions.

**Figure 6:**
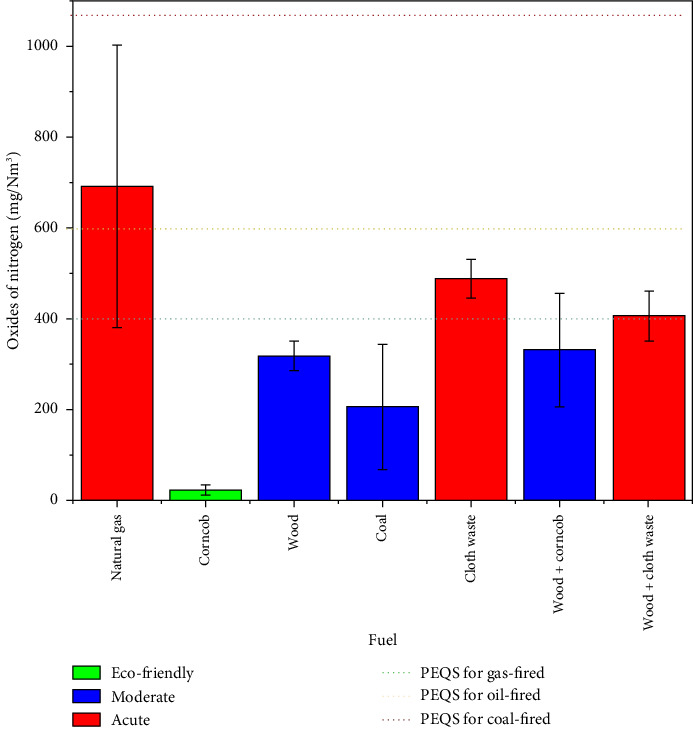
Comparison of oxides of nitrogen emissions across various fuel types relative to PEQS.

Figure 6 illustrates the variation of fuel with reference to emission and PEQS (represented by dotted lines) as follows: Natural gas emits the highest NO_x_ levels (∼700 mg/Nm^3^) and is classified as acute among the other fuels due to its high combustion temperatures, exceeding the gas-fired and oil-fired PEQS but still within the coal-fired PEQS. Compared to all PEQS limits, corncob has the lowest NO_x_ emissions (23 mg/Nm^3^). Wood (∼300 mg/Nm^3^) and wood + corncob (∼330 mg/Nm^3^) fall within the moderate category, staying well below all PEQS thresholds. Cloth waste (∼490 mg/Nm^3^) and wood + cloth waste (∼400 mg/Nm^3^) are classified as acute since they exceed gas-fired PEQS and approach oil-fired PEQS. Coal (∼200 mg/Nm^3^) is moderate, with emissions comfortably below all PEQS limits. Fuels like corncob and wood are cleaner alternatives, whereas cloth-based fuels require stricter emission controls.

### 3.6. Ranking of Environmentally Friendly Fuels

Natural gas is considered more environmentally friendly than coal or biomass due to its high hydrogen-to-carbon ratio (H/C), which produces more water vapors and less carbon dioxide (CO_2_). The absence of ash and other impurities results in minimal PM, making little sulfur oxide (SO_x_) emissions [36]. Despite high combustion temperatures, it generates moderate NO_x_ emissions, which can be controlled with advanced technologies. Generally speaking, natural gas is a sustainable energy choice because of its cleaner combustion chemistry and reduced pollutant output [38]. The current study is evident because of its low CO emissions (334.8 mg/Nm^3^), negligible SO_x_ emissions (175 mg/Nm^3^), and moderate NO_x_ emissions (692 mg/Nm^3^).

Wood and corncob are classified as moderate because they emit relatively higher CO (923.51–1172 mg/Nm^3^) and SO_x_ (1354–1418 mg/Nm^3^) emissions, although they emit NO_x_ (23–317 mg/Nm^3^) within PEQS limits. The combustion of mixed fuels, such as wood and corncob, reduces overall emissions through dilution effects [35] due to dilution effects during combustion. Coal, while also categorized as moderate, has higher variability in emissions, particularly SO_x_ (1902 mg/Nm^3^). Cloth waste and wood + cloth waste are classified as acute due to their high levels of SO_x_ (2208–2978 mg/Nm^3^) and NO_x_ (406–487 mg/Nm^3^), exceeding PEQS thresholds. The synthetic composition of these fuels causes inefficient combustion and high pollution levels, necessitating stricter regulations and advanced ECS.

**Figure 7:**
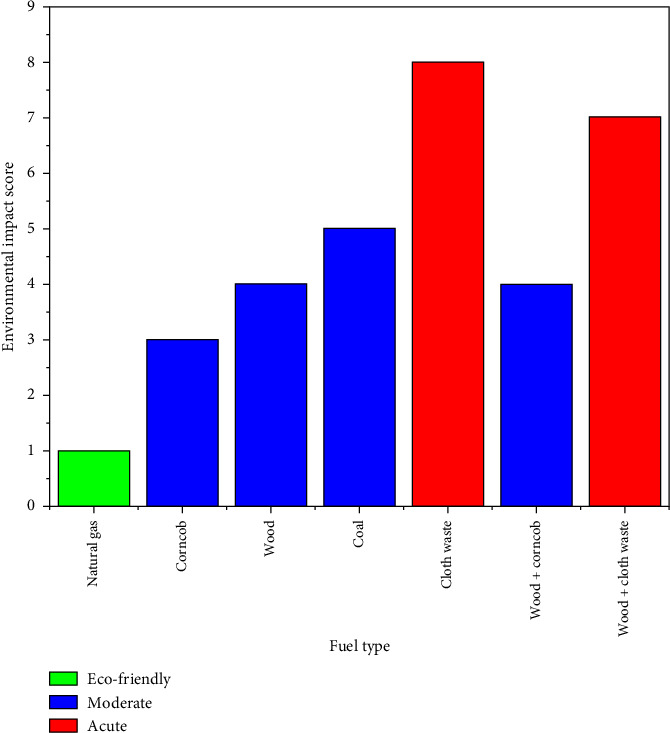
Environmental impact scores of the different fuels used in the study.

The overall environmental impact score is elaborated in Figure 7.

### 3.7. Comparative Analysis of Fuel Emissions in Compliance With PEQS and International Standards

The smoke opacity (measured as smoke number) from various fuel types, including natural gas, corncob, wood, coal, cloth waste, wood + corncob, and wood + cloth waste, is compared against the emission standards set by PEQS, US EPA, India, and China. The smoke opacity of natural gas is lower than all other fuels and complies with the strictest international standards. In contrast, coal and cloth waste show the highest smoke opacity, significantly exceeding the standards and indicating a high environmental impact. Wood and corncob produce moderate levels of smoke opacity, exceeding some standards but still cleaner than coal and cloth waste. The combinations of wood + corncob and wood + cloth waste offer cleaner alternatives to coal but still fail to meet the stricter standards of the US EPA and India. Natural gas is the most environmentally friendly option, while coal and cloth waste are the least suitable for reducing visible pollution.

Carbon monoxide emissions from different fuel types (natural gas, corncob, wood, coal, cloth waste, wood + corncob, and wood + cloth waste) against the emission standards set by PEQS, US EPA, India, and China. Natural gas emits the lowest levels of CO, well below both the US EPA limit (35.0 ppm) and the PEQS limit (800 mg/Nm^3^), making it the cleanest fuel in terms of CO emissions. Corncob, wood, coal, and cloth waste produce higher emissions, exceeding the PEQS and US EPA limits, with coal and cloth waste having the highest emissions. Wood + corncob and wood + cloth waste exceed the PEQS limit but produce slightly lower emissions than coal and cloth waste. Natural gas is the most environmentally friendly fuel, while coal and cloth waste are the least suitable due to their higher emissions.

Sulfur dioxide emissions from various fuel types (natural gas, corncob, wood, coal, cloth waste, wood + corncob, and wood + cloth waste) alongside SO_2_ standards are set by PEQS, the US EPA, India, and China. Natural gas emits the lowest levels of SO_2_, staying well within all regulatory limits, making it the cleanest fuel option. Corncob, wood, and cloth waste exceed the PEQS limit but remain within the standards of India and China. Coal and wood + cloth waste show the highest SO_2_ emissions, surpassing all international and local standards, indicating their high environmental impact. Natural gas stands out as the most environmentally friendly fuel, while coal and cloth waste are the least suitable due to their excessive emissions. Biomass fuels like wood and corncob are cleaner alternatives but require emission control to comply with PEQS.

Nitric oxide emissions from various fuel types (natural gas, corncob, wood, coal, cloth waste, wood + corncob, and wood + cloth waste) against the NO emission standards are set by PEQS, US EPA, India, and China. Natural gas has the highest NO emissions, exceeding the PEQS and China limits but staying within the US EPA and India standards. Corncob produces the lowest NO emissions and is well below all regulatory limits, making it the cleanest option. Wood, coal, and cloth waste show moderate emissions that fall below the PEQS limit but exceed the stricter US EPA, India, and China standards. Wood + corncob and wood + cloth waste produce higher emissions, exceeding the US EPA and India limits while staying below the PEQS and China standards. Corncob is the eco-friendliest fuel for NO emissions, while coal and cloth waste exceed several international limits, making them less suitable.

### 3.8. Recommendations on the Mitigation Strategies

It is imperative that the study incorporates mitigation strategies in order to address the harmful emissions generated by the textile industry in Faisalabad. These strategies include adopting advanced ECS to reduce air pollutants, switching to cleaner fuels like natural gas, implementing stricter environmental regulations aligned with PEQS, and upgrading to energy-efficient boiler systems. Furthermore, promoting carbon offsetting mechanisms and waste-to-energy solutions can further reduce environmental impact and promote sustainable practices. A shift to environmentally friendly fuels could be supported by providing government subsidies or incentives through carbon credit mechanisms.

## 4. Conclusion

This study identifies the environmental impacts of flue gas emissions from various fuel types used in the textile industry of Faisalabad, Pakistan. It categorizes into eco-friendly, moderate, and acute categories. Natural gas emerged as an eco-friendly fuel, complying fully with PEQS. At the same time, wood, corncob, and coal were classified as moderate due to their relatively higher emissions. Cloth waste and mixed fuels like wood and cloth waste were found to have the most acute environmental impacts. Based on the findings of the study, textile industries using coal, corncob, and cloth waste should install ECS to reduce the flue gas emissions to the atmosphere and keep the ambient air quality in safe ranges.

## Figures and Tables

**Table 1:** Standardized test methods for flue gas concentrations.

Parameters	Reference method
Oxides of sulfur	US EPA Method 6 c
Oxides of nitrogen	US EPA Method 7 e
Oxides of carbon	US EPA Method 3a and US EPA Method 10

**Table 2:** Classification of fuels based on source and composition.

Fuel category	Fuel	Description
Pure fuels	Natural gas (sui gas)	Fossil fuel
	Corncob	Biomass fuel
	Wood	Biomass fuel
	Coal	Fossil fuel
	Cloth waste (landa)	Industrial waste fuels

Mixed fuels	Wood + corncob	Mixed biomass
	Wood + cloth waste	Mixed biomass + industrial waste

**Table 3:** Concentrations of pollutants across fuel types.

Fuel types	Smoke opacity (smoke number)	Flue gases		
		Oxides of carbon (mg/Nm^3^)	Oxides of sulfur (mg/Nm^3^)	Oxides of nitrogen (mg/Nm^3^)
Natural gas	1.0 ± 0.000	334.8 ± 31.507	175.00 ± 95.247	692 ± 310
Corncob	3.2 ± 1.303	1132.0 ± 32.280	1418.00 ± 19.467	23 ± 11.74
Wood	3.6 ± 1.236	923.5 ± 83.948	1354.667 ± 61.319	317.33 ± 32.671
Coal	3.8 ± 1.0387	1355.2 ± 434.640	1902.147 ± 709.894	206.16 ± 137.404
Cloth waste	5.57 ± 1.272	2091.4 ± 194.111	2978.143 ± 93.1798	487.5 ± 41.603
Wood + corncob	4.0 ± 0.000	975.6 ± 63.610	1196.000 ± 571.800	331.16 ± 124.99
Wood + cloth waste	5.5 ± 1.732	1264.6 ± 133.665	2208.333 ± 354.323	406 ± 55.659

*Note:* Statistical significance means ± SD is based on a number of samples. Based on three samples analyzed individually in triplicate (*n* = 3 × 3). Significant differences (*p* < 0.05) were tested on monitored results.

**Table 4:** Smoke opacity and fuel rankings.

Fuel category	Fuels	Smoke opacity (smoke number)	Ranking
Pure fuels	Natural gas	1.0 ± 0.000	Eco-friendly
	Corncob	3.2 ± 1.303	Moderate
	Wood	3.6 ± 1.236	Moderate
	Coal	3.8 ± 1.0387	Moderate
	Cloth waste	5.0 ± 1.272	Acute

Mixed fuels	Wood + corncob	4.0 ± 0.000	Moderate
	Wood + cloth waste	4.1 ± 1.732	Acute

*Note:* Ranking categories are defined as eco-friendly (smoke number ≤ 2), moderate (smoke number > 2 and ≤ 4), and acute (smoke number > 4), with values presented as mean ± standard deviation (SD) based on three replicates (*n* = 3).

**Table 5:** Fuel types in comparison to oxides of carbon.

Fuel category	Fuels	CO (mg/Nm^3^)	Ranking
Pure fuels	Natural gas	334.8 ± 31.507	Eco-friendly
	Corncob	1132 ± 32.280	Moderate
	Wood	923.5 ± 83.948	Moderate
	Coal	1355.2 ± 434.640	Moderate
	Cloth waste	2091.4 ± 194.111	Acute

Mixed fuels	Wood + corncob	975.6 ± 63.610	Moderate
	Wood + cloth waste	1264.6 ± 133.665	Acute

*Note:* Ranking categories are defined as eco-friendly (CO ≤ 800 mg/Nm^3^), moderate (CO ≥ 1200–1400 mg/Nm^3^), and acute (CO ≥ 1500 mg/Nm^3^), with values presented as mean ± standard deviation (SD) based on three replicates (*n* = 3). The ranking of tested fuels with respect to CO (best to worst) was as follows: Natural gas > Wood > (Wood + corncob) > Corncob > Wood + cloth waste > Coal > Cloth waste.

**Table 6:** Fuel types in comparison to oxides of sulfur (SO_x_).

Fuel category	Fuels	SO_2_ (mg/Nm^3^)	Ranking
Pure fuels	Natural gas	175.00 ± 95.247	Eco-friendly
	Corncob	1418.00 ± 19.467	Moderate
	Wood	1354.667 ± 61.319	Moderate
	Coal	1902.147 ± 709.894	Moderate
	Cloth waste	2978.143 ± 93.1798	Acute

Mixed fuels	Wood + corncob	1196.000 ± 571.800	Moderate
	Wood + cloth waste	2208.333 ± 354.323	Acute

*Note:* Ranking categories are defined as eco-friendly (SO_2_ ≤ 1000 mg/Nm^3^), moderate (SO_2_ > 1000 mg/Nm^3^ and ≤ 2000 mg/Nm^3^), and acute (SO_2_ > 2000 mg/Nm^3^), with values presented as mean ± standard deviation (SD) based on three replicates (*n* = 3). The ranking for best fuel can be as follows: Natural gas > (Wood + corncob) > Wood > Corncob > Coal > (Wood + cloth waste) > Cloth waste.

**Table 7:** Fuel types in comparison to oxides of nitrogen (NO_x_).

Fuel category	Fuels	NO_x_ (mg/Nm^3^)	Ranking
Pure fuels	Natural gas	692 ± 310	Acute
	Corncob	23 ± 11.74	Eco-friendly
	Wood	317.33 ± 32.671	Moderate
	Coal	206.16 ± 137.404	Moderate
	Cloth waste	487.5 ± 41.603	Acute

Mixed fuels	Wood + corncob	331.16 ± 124.99	Moderate
	Wood + cloth waste	406 ± 55.659	Acute

*Note:* Fuel categories are defined as eco-friendly (NO_x_ ≤ 100 mg/Nm^3^), moderate (NO_x_ > 100 mg/Nm^3^ and ≤ 400 mg/Nm^3^), and acute (NO_x_ > 400 mg/Nm^3^). Emission values are presented as mean ± standard deviation (SD) based on three replicates (*n* = 3). Statistical significance was tested at *p* < 0.05.

## Data Availability

The data that support the findings of this study are available from the corresponding author upon reasonable request.

## Nomenclature

AQ: Air quality
AQI: Air Quality Index
CO: Carbon monoxide
CO_2_: Carbon dioxide
EPA: Environmental Protection Agency
EP&CCD: Environmental Protection and Climate Change Department
ECS: Emission control system
GHGs: Greenhouse gases
HC: Hydrocarbons
ICA: Intercity area
LSSVM: Least squares support vector machines
mg/Nm^3^: Milligram per normal cubic meter
MIE: Millat Industrial Estate
NAMA: National Appropriate Mitigation Actions
NO_x_: Oxides of nitrogen
SO_x_: Oxides of sulfur
O_3_: Ozone
PM: Particulate matter
PEQS: Punjab Environmental Quality Standards
RS: Ringelmann Scale
SIE: Small Industrial Estate
SVR: Support vector regression
SDGs: Sustainable development goals
TSP: Total suspended particles
US EPA: United States Environmental Protection Agency
VOCs: Volatile organic compounds
WHO: World Health Organization

## References

[1] Beavan A., Härtel S., Spielmann J., Koehle M. (2023). Air Pollution, a Worthy Opponent? How Pollution Levels Impair Athlete Performance Across Physical, Technical, and Cognitive Domains. *Science of the Total Environment*.

[2] Font-Ribera L., Rico M., Marí-Dell’Olmo M., Oliveras L., Trapero-Bertran M., Pérez (2023). Estimating Ambient Air Pollution Mortality and Disease Burden and Its Economic Cost in Barcelona. *Environmental Research*.

[3] Poulsen A. H., Hvidtfeldt U. A., Sørensen M. (2023). Air Pollution With NO2, PM2. 5, and Elemental Carbon in Relation to Risk of Breast Cancer–A Nationwide case-control Study from Denmark. *Environmental Research*.

[4] Al-Taai, Hassan S. H., al-Dulaimi M., Abood W. (2022). Air Pollution: a Study of Its Concept, Causes, Sources and Effects. *Asian Journal of Water Environment and Pollution*.

[5] Venkatesan P. (2016). WHO Report: Air Pollution is a Major Threat to Health. *The Lancet Respiratory Medicine*.

[6] Dominski F. H., Lorenzetti Branco J. H., Buonanno G., Stabile L., Gameiro da Silva M., Andrade A. (2021). Effects of Air Pollution on Health: a Mapping Review of Systematic Reviews and meta-analyses. *Environmental Research*.

[7] Islam S., Islam S., Akm S. I., Urmy Z., Ahmed S., Islam A. (2020). A Study on the Solutions of Environment Pollutions and Worker’s Health Problems Caused by Textile Manufacturing Operations. *Biomedical Journal of Scientific & Technical Research*.

[8] Zuberi M. J. S., Shehabi A., Rao P. (2024). Cross-Sectoral Assessment of CO2 Capture from US Industrial Flue Gases for Fuels and Chemicals Manufacture. *International Journal of Greenhouse Gas Control*.

[9] Węgiel M., Chrząszcz R., Maślanka A., Grochowalski A. (2014). Study on the Impact of Industrial Flue Gases on the PCDD/Fs Congener Profile in Ambient Air. *Chemosphere*.

[10] Gaffney J. S., Marley N. A. (2009). The Impacts of Combustion Emissions on Air Quality and climate–from Coal to Biofuels and Beyond. *Atmospheric Environment*.

[11] Munawer M. E. (2021). Human Health and Environmental Impacts of Coal Combustion and Post-combustion Wastes. *Journal of Sustainable Mining*.

[12] Tomlin A. S. (2021). Air Quality and Climate Impacts of Biomass Use as an Energy Source: a Review. *Energy & Fuels*.

[13] Mehmood K. A., Raza H., Abid A., A., Guo, Ping (2018). A Preliminary Assessment and Control Strategy of Size Segregated Pollutants in Urban and peri-urban Areas of Metropolitan Faisalabad, Pakistan. *Pakistan Journal of Analytical & Environmental Chemistry*.

[14] Nasir A., Aslam R. A., Ali F., Nasir A. (2024). Air Pollution from Industrial Emissions and Its Control in Pakistan: Current Situation, Challenges, and Way Forward.

[15] Khan M. M., Muhmood K., Mahmood H. Z., Khaliq I. H., Zaman S., Zaman (2024). The Health and Economic Burden of Dust Pollution in the Textile Industry of Faisalabad, Pakistan. *Journal of the Egyptian Public Health Association*.

[16] Yasmeen R., Shah W. U. H., Ivascu L., Tao R., Sarfraz M. (2022). Energy Crisis, Firm Productivity, Political Crisis, and Sustainable Growth of the Textile Industry: an Emerging Economy Perspective. *Sustainability*.

[17] Shahid K. M. A., Hussain K. (2015). A Study of Air Pollution and Human Health in Faisalabad City, Pakistan. *International Journal of Core Engineering and Management*.

[18] Fatima M., Butt I., Nasar-u-Minallah M., Atta A., Cheng G. (2023). Assessment of Air Pollution and Its Association With Population Health: Geo-Statistical Evidence from Pakistan. *Geography, Environment, Sustainability*.

[19] Tabinda B., A., Habib, Abdullah Rasheed R. (2020). Ambient Air Quality of Faisalabad With Relevance to the Seasonal Variations. *MAPAN-journal of Metrology Society of India*.

[20] Javed N. Q. (2019). City Profile: Faisalabad, Pakistan. *Environment and Urbanization ASIA*.

[21] Dieng T., M., Iwanaga T., Y., Christie Y. (2020). Evaluation of Performance and Emission Characteristics of Biodiesel Fuel Produced from Rapeseed Oil. *Journal of Energy and Power Engineering*.

[22] Zubair M., Farid M., Danish M., Zafar M. N. (2017). Evaluation of Air Pollution Sources in Selected Zone of Textile Industries in Pakistan. *Environmental Engineering and Management Journal*.

[23] Thi Hong Phuong P., Trung Dung N., Thi Mai Thao P., Tham T. T. (2022). Emissions Factors of Air Pollutants from Rice Straw burning-hood Experiments. *VNU Journal of Science: Earth and Environmental Sciences*.

[24] Chen C., Yao A., Yao C. (2019). Study of the Characteristics of PM and the Correlation of Soot and Smoke Opacity on the Diesel Methanol Dual Fuel Engine. *Applied Thermal Engineering*.

[25] Khurshid I., Ahmad S., Nawaz R. (2018). Development of Fire Bricks from Organic Waste: an eco-friendly Energy Solution. *Applied Ecology and Environmental Research*.

[26] Malhi M., H., Ahmed I., N., Iqra, Haider R. (2023). Monitoring of Ambient Air Pollution in Lahore City. *Pakistan Journal of Emerging Sciences and Technologies*.

[27] Putra S. D., Fernandez D. (2018). Optimization of Digital Image Processing Method to Improve Smoke Opacity Meter Accuracy. *International Journal on Informatics Visualization*.

[28] Nazar M., Yasar A., Raza S. A. (2021). Techno-Economic and Environmental Assessment of Rice Husk in Comparison to Coal and Furnace Oil as a Boiler Fuel. *Biomass Conversion and Biorefinery*.

[29] Sugeng, Avianto D., Yahya J., W., Muhsin A., Kusdi (2020). Experimental Comparison of Smoke Opacity and Particulate Matter Emissions With the Use of Emulsion Fuel. *Kyushu University Institutional*.

[30] Boqijonov F., Nazirova M. (2024). Hygienic Assessment of the Level of Atmospheric Air Pollution. *Ethiopian International Journal of Multidisciplinary Research*.

[31] Giakoumis E. G., Rakopoulos C. D., Dimaratos A. M., Rakopoulos D. C. (2013). Exhaust Emissions With Ethanol or n-butanol Diesel Fuel Blends During Transient Operation: a Review. *Renewable and Sustainable Energy Reviews*.

[32] Vicente E., Duarte M., Calvo A., Nunes T., Tarelho L., Alves C. (2015). Emission of Carbon Monoxide, Total Hydrocarbons and Particulate Matter During Wood Combustion in a Stove Operating Under Distinct Conditions. *Fuel Processing Technology*.

[33] Cheng Y., Hongqiang M., Hongyu C. (2018). Preparation and Characterization of Coal Gangue Geopolymers. *Construction and Building Materials*.

[34] Liu X., Chen M., Wei Y. (2015). Combustion Behavior of corncob/bituminous Coal and hardwood/bituminous Coal. *Renewable Energy*.

[35] Liu L., Memon Z. M., Xie Y. (2023). Recent Advances of Research in Coal and Biomass co-firing for Electricity and Heat Generation. *Circular Economy*.

[36] Sharma B., Kuttippurath J., Patel V., Gopikrishnan G. (2024). Regional Sources of NH_3_, SO_2_ and CO in the Third Pole. *Environmental Research*.

[37] Pronobis M., Wejkowski R. (2023). Conversion of a Pulverized Coal Boiler into a Torrefied Biomass Boiler. *Energy*.

[38] Farooq M. T., Venkatachalapati A., Manzoor Z. (2024). Comprehensive Characterization of Unscientifically Disposed Municipal Solid Waste (MSW) in Kashmir Region, India. *Environmental Monitoring and Assessment*.

[39] Chen X., Zhang H., Xing X., Qin H. (2021). Modeling NOx Emissions With an Intelligent Combinatorial Algorithm. *Mathematical Problems in Engineering*.

